# *FGFR1* Amplification Is Often Homogeneous and Strongly Linked to the Squamous Cell Carcinoma Subtype in Esophageal Carcinoma

**DOI:** 10.1371/journal.pone.0141867

**Published:** 2015-11-10

**Authors:** Katharina von Loga, Jule Kohlhaussen, Lia Burkhardt, Ronald Simon, Stefan Steurer, Susanne Burdak-Rothkamm, Frank Jacobsen, Guido Sauter, Till Krech

**Affiliations:** Department of Pathology, University Medical Center Hamburg-Eppendorf, Hamburg, Germany; Advanced Centre for Treatment, Research and Education in Cancer, Tata Memorial Center, INDIA

## Abstract

**Background and Aims:**

Amplification of the *fibroblast growth factor receptor 1* (*FGFR1*) is believed to predict response to multi-kinase inhibitors targeting *FGFR1*. Esophageal cancer is an aggressive disease, for which novel targeted therapies are highly warranted.

**Methods:**

This study was designed to investigate the prevalence and clinical significance of *FGFR1* amplification in a tissue microarray containing 346 adenocarcinomas and 254 squamous cell carcinomas of the esophagus, using dual-labeling fluorescence *in situ* hybridization (FISH) analysis.

**Results:**

*FGFR1* amplification, defined as a ratio of *FGFR1*:centromere 8 copy numbers ≥ 2.0, was more frequently seen in squamous cell carcinoma (8.9% of 202 interpretable cases) than in adenocarcinoma (1.6% of 308; p<0.0001). There was no association between *FGFR1* amplification and tumor phenotype or clinical outcome. To study potential heterogeneity of *FGFR1* amplification, all available tumor blocks from 23 *FGFR1* amplified tumors were analyzed on conventional large sections. This analysis revealed complete homogeneity of *FGFR1* amplification in 20 (86.9%) primary tumors and in all available lymph node metastases. Remarkably, *FGFR1* amplification was also seen in dysplasia adjacent to tumor in 6 of 9 patients with *FGFR1* amplified primary cancers.

**Conclusions:**

In conclusion, *FGFR1* amplification occurs in a relevant subgroup of carcinomas of the esophagus and may play a particular role for development of squamous cell cancers. The high homogeneity of *FGFR1* amplification suggests that patients with *FGFR1* amplified esophageal cancers may particularly benefit from anti-*FGFR1* therapies and prompt for clinical studies in this tumor type.

## Introduction

Esophageal cancer is an aggressive disease presenting with two histologically and genetically distinct subtypes, i.e. adeno- and squamous cell carcinoma (EADC and ESCC). Patients with esophageal neoplasias are usually diagnosed at advanced stages [1,2] and, thus, have a generally poor prognosis with 5-year survival rates typically not extending 10–25% (URL: http://www.cancer.org) [1,3]. Because curative therapy options in patients with advanced disease are lacking, there is an urgent need for novel and effective drugs.

Targeted cancer therapies have successfully entered clinical routine in several tumor types. Particularly growth factor receptors, such as *HER2*, *EGFR*, *VEGFR or c-KIT*, which are strongly up regulated in many cancers, have proven to represent efficient anti-cancer therapy targets [4–10]. There is growing evidence that targeting of the fibroblast growth factor receptor 1 (FGFR1) holds promising clinical potential [11,12]. FGFR1 plays an important role in cell differentiation and growth by downstream signaling to the nucleus involving either the Ras/MAPK- or PI3/Akt-pathways [13,14]. An important mechanism of oncogenic FGFR1 activation is amplification of its gene locus at chromosome 8p11, which is found in 10–20% of squamous cell carcinomas of the lung [15–18], in about 10% of hormone receptor positive breast carcinoma [19–21], 10–17% head and neck squamous cell carcinomas [22] and 6% of small cell carcinomas of the lung [23]. Little is known about the clinical significance of *FGFR1* amplification in esophageal cancer or about possible differences between histological subtypes. Reported *FGFR1* amplification frequencies in studies on 32–189 esophageal cancers range between 6–21% in squamous cell cancers [24,25] and 9% in adenocarcinomas [25], but the impact on patient prognosis is largely unknown. Only one study on Asian ESCC patients suggested that *FGFR1* amplification might be linked to poor outcome [26].

To better understand the prognostic role of *FGFR1* amplification in Caucasian patients, we employed fluorescence in-situ hybridization (FISH) analysis for precise determination of the *FGFR1* amplification rate in a large tissue microarray made from 254 ESCC and 346 EADC patients with histopathological and clinical follow-up data of Caucasian origin.

## Material and Methods

### Esophageal cancer TMA

The esophageal cancer TMA utilized for this study consists of 600 formalin-fixed paraffin-embedded tissue samples including 346 esophageal adenocarcinomas and 254 esophageal squamous cell carcinomas, and was extended based on an earlier TMA containing 292 cancers [27]. All patients had undergone surgery between 1992–2011 at the surgical department of the University Medical Center Hamburg-Eppendorf. The female to male ratio in our cancers was 117 to 483, which corresponds to the observed incidence of these tumor types [28,29]. Two pathologists (KVL, TK) reviewed all tumor slides. All work has been carried out in compliance with the Helsinki Declaration. The general usage of archived diagnostic left-over tissues for manufacturing of tissue microarrays (TMAs) and their analysis for research purposes as well as patient data analysis has been approved by the local ethics committee (Ethics commission Hamburg, WF-049/09 and PV3652). The authors KVL and FJ acted as the treating physicians/pathologists and had access to identifying patient information at the time point when the tissues were collected but not at the time point when the study was conducted. The tissues were collected during routine cancer surgery. All tissues had been collected and used for TMA manufacturing prior to this study. The ethics committee reviewed and approved the lack of consent procedure.

The TMA manufacturing process was described earlier in detail [30]. In short, one 0.6 mm core was taken from a representative tissue block from each patient. Tissue sample were distributed on two TMA blocks, containing 346 and 254 cancer cores, respectively. In addition, both blocks comprise tissue controls of normal esophageal epithelium. Tumor grade and stage were defined according to the International Union Against Cancer (UICC) and the WHO [3,31]. Clinical data of patients were retrospectively evaluated. The medium follow-up period was 27, 7 months (range 0–215 months). An overview of all histological and clinical data is given in Table 1.

**Table 1:** Esophageal Carcinoma—Array.

		ESCC (n = 254)	EADC (n = 346)
**Gender**	female	69	48
	male	185	298
**Tumor**	pT1a	16	33
	pT1b	34	49
	pT2	49	36
	pT3	139	206
	pT4a	6	18
	pT4b	10	4
**Nodal**	pNX	9	8
	pN0	116	107
	pN1	56	63
	pN2	48	80
	pN3	25	88
**Metastasis**	pM0	206	307
	pM1	48	39
**Grade**	G1	4	26
	G2	164	125
	G3	86	189
	G4	0	6
**UICC**	IA	40	71
	IB	24	12
	IIA	46	29
	IIB	13	14
	IIIA	35	57
	IIIB	26	54
	IIIC	22	72
	IV	48	37

### Fluorescence *in situ* hybridization (FISH)

A dual color FISH probe set was used for *FGFR1* amplification analysis. The probe set combined a home-made spectrum green labeled *FGFR1* probe (chromosome 8 locus 8p 11.22–23, made from bacterial artificial chromosome (BAC) clone RP11-350N15) and a commercial spectrum red labeled probe for the centromeric region of chromosome 8 (Zytovision, Bremerhaven, Germany). Freshly cut TMA sections (4 micrometer thick) were deparaffinized and proteolytically pretreated using a commercial kit (Zytolight FISH-Tissue Implementation Kit, Zytovision, Bremerhaven, Germany), followed by dehydration in 70%, 90% and 99% ethanol, air drying and codenaturation in a Thermobrite hybridization oven (Abbott, Chicago, USA) for 10 minutes at 75°Celsius. Hybridization was overnight at 37°Celsius. Slides were then washed and counterstained with 0, 2 micromol/l of DAPI.

### Scoring of FISH signals

Presence of tumor cells was verified in each spot by comparison of a hematoxilin and eosin (H&E) stained adjacent reference section of the TMA. Two experienced technicians (SS, SE) estimated the predominant gene and centromere copy numbers in at least 20 non-overlapping tumor cells in each tissue spot. Data from our laboratory have previously shown that diagnosis of amplification based on signal number estimation is highly reliable [32,33].

**Fig 1:**
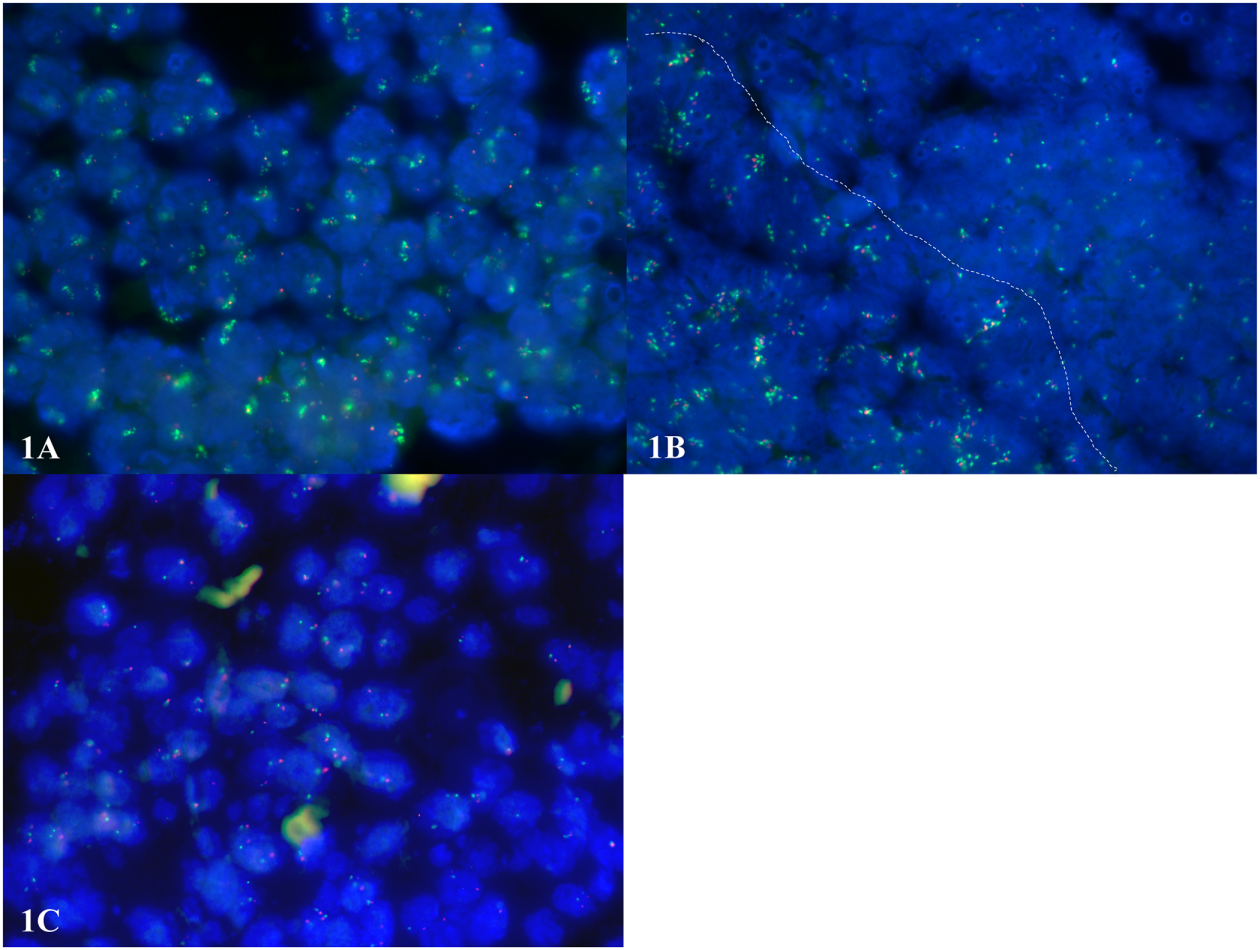
FISH analysis in ESCC patients. Green signals represent the *FGFR1* gene while red signals correspond to the centromere of chromosome 8. (A) high-level amplification of *FGFR1* showing 10–20 gene signals and 2–4 centromere signals with a ratio of 6.16. (B) Heterogeneous amplification of *FGFR1* as indicated by presence of two distinct cancer areas with FGFR1 amplification and without FGFR1 amplification. These two areas are separated by the dotted line. (C) Normal *FGFR1* gene and centromere 8 signals.

High-level FGFR1 amplification was defined as presence of ≥10 FGFR1 gene signals or an *FGFR1*/centromere 8 ratio of ≥3.0. Tumors with a ratio of ≥2.0 but <3.0 were considered low-level amplification. All other cancers were considered non-amplified. These included cancers with normal (two) copies of *FGFR1* and centromere 8, cancers with polyploidy of chromosome 8 (ratio >0.8 but <1.2 and more than two *FGFR1* copies) as well as cancers with an *FGFR1* copy number gain not reaching the threshold for amplification (ratio ≥1.2 but <2.0). Examples of tumor spots with and without *FGFR1* amplification are shown in Fig 1 (Fig 1).

### Large section validation

To estimate the degree of intratumoral heterogeneity of *FGFR1* amplification, all available primary and metastasis tumor blocks of all cancers showing *FGFR1* amplification according to the TMA analysis, including 18 ESCC and 5 EADC, were analyzed for amplification on conventional large sections (4 μm thickness). The number of *FGFR1* and centromere 8 FISH signals were counted in at least 20 non-overlapping cell nuclei, and the average *FGFR1* and centromere 8 copy numbers were calculated per sample. The *FGFR1*:centromere 8 ratio was calculated from these values. High-level and low-level *FGFR1* amplification was defined as described above. Heterogeneity was defined as presence of *FGFR1* non-amplified and amplified tumor areas within the same cancer (Fig 1B). If present, adjacent dysplasia was also evaluated.

For comparison of FGFR1 expression levels in tumors with and without *FGFR1* amplification, tissue blocks containing 70% or more tumor cells were selected that had been used for TMA manufacturing before. For RNA isolation, one 0.6 mm tissue core was taken from each tumor block. The deparaffinized and air-dried cores were grinded in liquid nitrogen before total RNA was isolated using a commercial kit (RNeasy FFPE kit #744044, QIAGEN) following the manufacturers instructions except for prolonged (overnight) proteinase digestion. cDNA was synthesized from 0.5 to 1 mg total RNA using the High Capacity cDNA Reverse Transcription Kit (Applied Biosystems #4368814). Quantitative reverse transcriptase PCR (qRT-PCR) was carried out in duplicate using combinations of primer pairs and TaqMan probes targeting mRNA sequences of FGFR1 and glyceraldehyde-3-phosphate dehydrogenase (GAPDH). Primers were obtained from Applied Biosystems (Darmstadt). The GAPDHgene served as an internal control for the normalization of FGFR1 RT-PCR products. The PCR program included a 10 minute denaturation at 95_C followed by 40 cycles of 15 seconds at 95_C, and 1 minute at 60_C. Relative quantification results were calculated according to the DDCt method [34].

### Statistical analysis

Statistical calculations were performed using SAS’ JMP (version 9.0) statistical software. To compare categorical variables such as grade, stage and molecular features, contingency tables were calculated applying chi^2^-test and Fisher’s exact tests. Survival curves were calculated according to the Kaplan-Meier method and compared with the Logrank test. Cox regression was used to assess independency of molecular, morphological and clinical parameters to predict patient survival.

## Results

### Technical aspects

FISH analysis was successful in 510 of 600 (85%) arrayed tumors including 202 squamous cell carcinomas and 308 adenocarcinomas. Analysis failures were either due to insufficient hybridization efficiency or issues connected to the TMA technology, such as missing samples or absence of unequivocal cancer cells in a tissue spot.

### Prevalence of *FGFR1* amplifications and their association to esophageal cancer phenotype and patient prognosis

**Fig 2:**
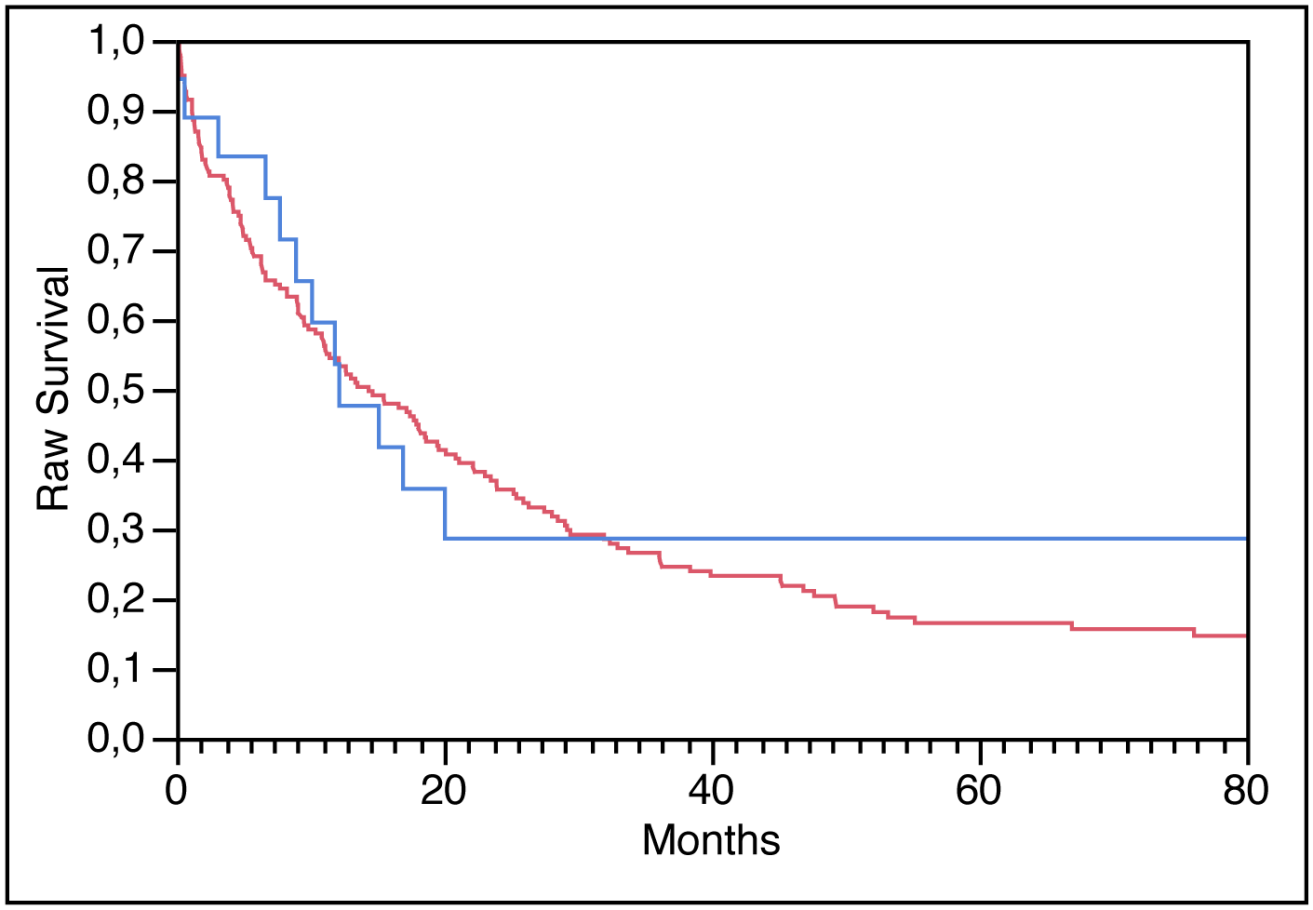
Raw Survival of ESCC patients. Red line: no *FGFR1* amplified tumor patients. Blue line: *FGFR1* amplified tumor patients.

Low- and high-level *FGFR1* amplifications were significantly more frequent in ESCC (18 of 202, 8.9%) than in EADC (5 of 308, 1.6%, p<0.0001). *FGFR1* amplification was typically high-level according to our predefined criteria: 67% (12 of 18) *FGFR1*-amplified ESCC, and 80% (4 of 5) *FGFR1*-amplified EADC showed high-level amplification. Due to these strong differences in amplification frequencies between squamous cell- and adenocarcinomas, associations to phenotype and clinical outcome were calculated separately in each subgroup. All results are summarized in Table 2. These analyses did not reveal significant associations between *FGFR1* amplification and tumor phenotype or clinical outcome, neither in the subset of 202 ESCC, nor in the subset of 308 EADC (Fig 2). A total of 21 squamous cell carcinomas and 4 adenocarcinomas harbored *FGFR1* copy number increases that did not reach the predefined threshold for amplification, including 15 cancers with polyploidy of chromosome 8 (15 ESCC) and 10 cancers with *FGFR1* gains (6 ESCC and 4 EADC). The results of these cases are shown in S1 Table.

**Table 2:** *FGFR1* ampification in ESCC and EADC.

		ESCC					EADC				
		n	all ampl. (%)	low ampl. (%)	high ampl. (%)	p-value	n	all ampl. (%)	low ampl. (%)	high ampl. (%)	p-value
**all cancers**		202	18 (8.9)	6 (3.0)	12 (5.9)		308	5 (1.6)	1 (0.3)	4 (1.3)	
**Gender**	female	53	4 (7.5)	1 (1.9)	3 (5.7)		41	1 (2.4)	0	1 (2.4)	
	male	149	14 (9.4)	5 (3.4)	9 (6.0)	0.8357	267	4 (1.5)	1 (0.4)	3 (1.1)	0.733
**Tumor**	pT1a	9	1 (11.1)	0	1 (11.1)		29	0	0	0	
	pT1b	27	3 (11.1)	0	3 (11.1)		44	1 (2.3)	0	1 (2.3)	
	pT2	38	4 (10.5)	2 (5.3)	2 (5.3)		27	1 (3.7)	0	1 (3.7)	
	pT3	116	9 (7.8)	4 (3.4)	5 (4.3)		188	3 (1.6)	1 (0.5)	2 (1.1)	
	pT4a	5	1 (20.0)	0	1 (20.0)		16	0	0	0	
	pT4b	7	0	0	0	0.6832	4	0	0	0	0.9748
**Nodal**	pNX	4	0	0	0		8	0	0	0	
	pN0	95	8 (8.4)	3 (3.2)	5 (5.3)		96	3 (3.1)	0	3 (3.1)	
	pN1	45	4 (8.9)	1 (2.2)	3 (6.7)		54	0	0	0	
	pN2	41	5 (12.2)	1 (2.4)	4 (9.8)		71	1 (1.4)	0	1 (1.4)	
	pN3	17	1 (5.9)	1 (5.9)	0	0.7451	79	1 (1.3)	1 (1.3)	0	0.2219
**Metastasis**	pM0	165	12 (7.3)	4 (2.4)	8 (4.8)		273	5 (1.8)	1 (0.4)	4 (1.5)	
	pM1	37	6 (16.2)	2 (5.4)	4 (10.8)	0.2525	35	0	0	0	0.5543
**Grade**	G1	3	0	0	0		23	0	0	0	
	G2	136	13 (9.6)	4 (2.9)	9 (6.6)		111	0	0	0	
	G3	63	5 (7.9)	2 (3.2)	3 (4.8)	0.9308	174	5 (2.9)	1 (0.6)	4 (2.3)	0.2528
**UICC**	IA	28	2 (7.1)	0	2 (7.1)		63	1 (1.6)	0	1 (1.6)	
	IB	22	1 (4.5)	0	1 (4.5)		11	1 (9.1)	0	1 (9.1)	
	IIA	40	4 (10.0)	2 (5.0)	2 (5.0)		28	1 (3.6)	0	1 (3.6)	
	IIB	9	2 (22.2)	1 (11.1)	1 (11.1)		10	0	0	0	
	IIIA	27	0	0	0		50	0	0	0	
	IIIB	25	2 (8.0)	1 (4.0)	1 (4.0)		49	1 (2.0)	0	1 (2.0)	
	IIIC	14	1 (7.1)	0	1 (7.1)		63	1 (1.6)	1 (1.6)	0	
	IV	37	6 (16.2)	2 (5.4)	4 (10.8)	0.4709	34	0	0	0	0.6935

### Heterogeneity analysis

All 23 amplified cancers were further analyzed in order to assess the level of homogeneity/heterogeneity of *FGFR1* amplifications. Data are summarized in Table 3. Overall, *FGFR1* amplification was homogenous in 20 (86.9%) and heterogeneous in 3 (13.0%) amplified cancers. All available lymph node metastasis (n = 7) showed a homogeneous amplification pattern, even in one case with heterogeneous amplification of the primary tumor. Remarkably, *FGFR1* amplification was also observed in 6 of 8 patients (75%) where areas of dysplastic squamous epithelium were found adjacent to invasive cancer.

**Table 3:** Homogeneity/Heterogeneity analysis of *FGFR1* amplified tumors.

									*FGFR1* in the PT				*FGFR1* in the LN					
N°	Sub-type	Age	Gender	pT	pN	pM	G	UICC	*FGFR1* gene*	Cen 8*	*FGFR1* Ratio	Homo/Hetero**	*FGFR1* gene*	Cen 8*	*FGFR1* Ratio	PT	LN	Dys
1	ESCC	76	m	1b	1	1	2	IV	35.55	3.35	10.6	homogeneous				Ampl, high		Ampl
2	ESCC	62	m	1b	2	1	2	IV	35.5 + 3.2	4.1+ 3.15	8.55 + 1.02	heterogeneous	21.25	3.05	6.97	Ampl, high	Ampl	
3	ESCC	71	m	2	1	0	2	IIB	17.25	2.8	6.16	homogeneous	19.5	2.15	9.07	Ampl, high	Ampl	Ampl
4	ESCC	53	m	3	2	1	3	IV	15.2	2.3	6.61	homogeneous	9.25	2.35	3.94	Ampl, high	Ampl	
5	ESCC	71	m	3	1	1	2	IV	14.3	2.75	5.20	homogeneous				Ampl, high		
6	ESCC	53	m	3	0	0	2	IIA	14.0	2.35	5.96	homogeneous				Ampl, high		
7	ESCC	56	m	3	0	0	3	IIA	10.85	4.25	2.55	homogeneous				Ampl, high		
8	ESCC	53	f	4a	2	0	3	IIIC	10.45	3.2	3.27	homogeneous	9.05	3.2	2.83	Ampl, high	Ampl	Ampl
9	ESCC	52	m	2	0	0	2	IB	10.3	3.1	3.32	homogeneous				Ampl, high		
10	ESCC	61	f	3	2	0	2	IIIB	9.5	2.5	3.80	homogeneous	5.75	2.1	2.74	Ampl, high	Ampl	no Ampl
11	ESCC	60	f	1b	0	0	2	IA	8.4	2.45	3.43	homogeneous				Ampl, high		Ampl
12	ESCC	70	m	1a	0	0	2	IA	6.7	2.05	3.27	homogeneous				Ampl, high		Ampl
13	ESCC	47	m	2	0	1	2	IV	7.6 + 3.4	2.7 + 2.95	2.76 + 1.15	heterogeneous				Ampl, low		
14	ESCC	66	m	3	0	0	2	IIA	6.3 + 2.55	2.3 + 2.25	2.74 + 1.13	heterogeneous				Ampl, low		no Ampl
15	ESCC	71	m	3	2	0	3	IIIB	9.5	4.0	2.38	homogeneous	12.25	2.4	5.10	Ampl, low	Ampl	Ampl
16	ESCC	42	m	2	1	0	3	IIB	9.35	3.5	2.67	homogeneous	11.05	4.5	2.46	Ampl, low	Ampl	
17	ESCC	62	m	3	3	1	2	IV	5.15	2.3	2.24	homogeneous				Ampl, low		
18	ESCC	67	f	3	0	0	2	IIA	5.05	2.0	2.53	homogeneous				Ampl, low		
19	EADC	62	f	3	0	0	3	IIA	30.75	5.05	6.09	homogeneous				Ampl, high		
20	EADC	60	m	3	2	0	3	IIIB	18.5	5.35	3.46	homogeneous				Ampl, high		
21	EADC	56	m	2	0	0	3	IB	9.85	3.0	3.28	homogeneous				Ampl, high		
22	EADC	80	m	1b	0	0	3	IA	8.63	2.38	3.63	homogeneous				Ampl, high		
23	EADC	73	m	3	3	0	3	IIIC	6.25	2.85	2.19	homogeneous				Ampl, low		

*average copy number counted in 20 cell nuclei,

**Homogeneity/Heterogeneity, PT: primary tumor, LN: lymph node, Dys: dysplasia

### Association between *FGFR1* gene amplification and mRNA expression

**Fig 3:**
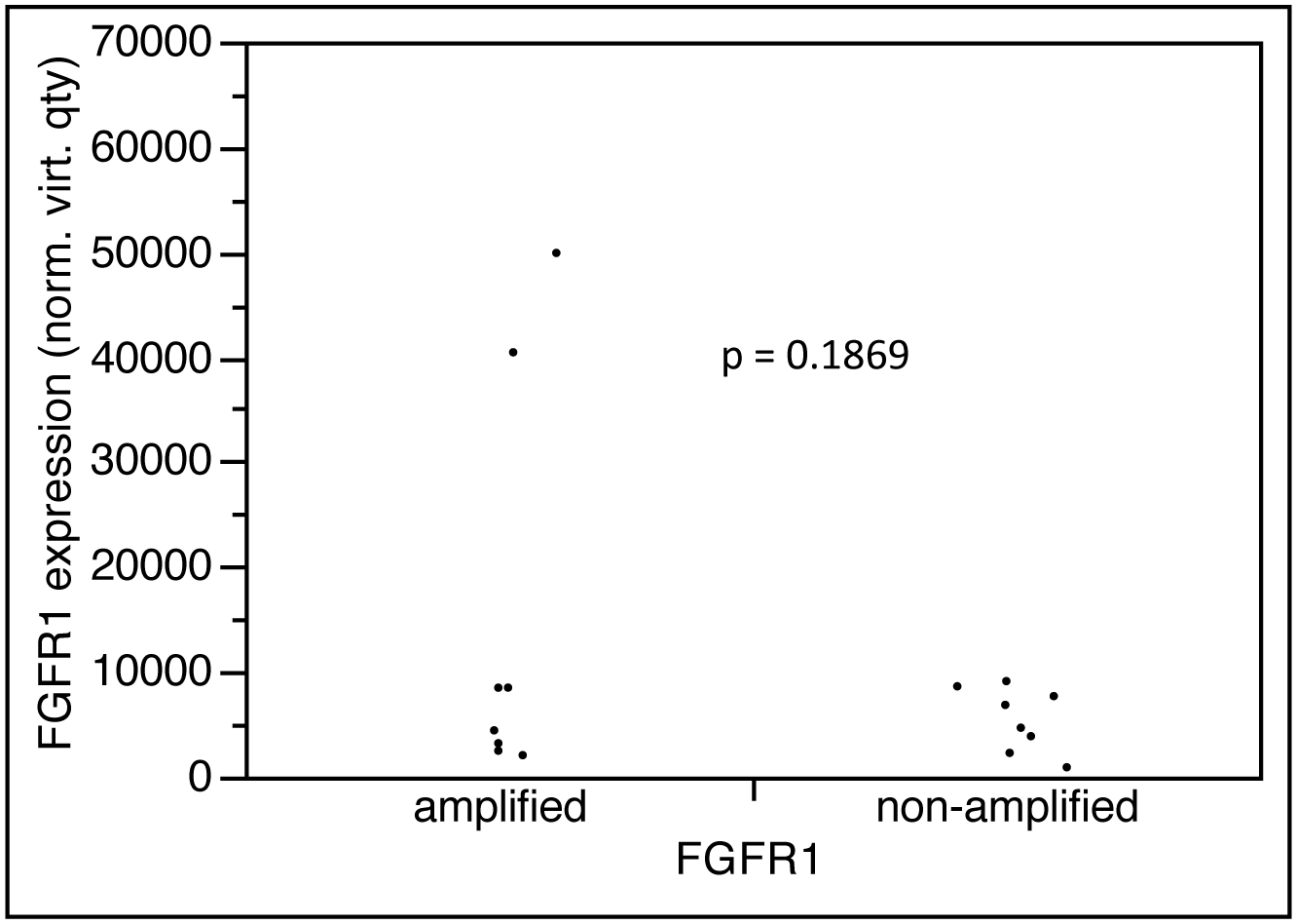
FGFR1 expression.

mRNA expression results were retained from 8 of 10 *FGFR1* amplified and *FGFR1* non-amplified each. The average mRNA expression level was 14919.4 in *FGFR1* amplified as compared to 5485.8 in *FGFR1* non-amplified (p = 0.1869). Of note, most *FGFR1* amplified cancers had expression levels that were in the range of *FGFR1* non-amplified samples. Highest expression levels were found in two amplified samples harboring 9.35 and 35.55 *FGFR1* gene copies according to FISH analysis (Fig 3).

## Discussion

Our data demonstrate marked differences in the prevalence of *FGFR1* gene amplification between squamous cell carcinomas and adenocarcinomas of esophageal carcinomas.

In this study, we employed FISH analysis for *FGFR1* gene copy analysis. FISH is regarded as the most precise means for gene copy number measurement in histological sections, because it is not disturbed by the presence of non-cancerous cells in the tissue samples. Previous studies on Caucasian ESCC employing the less quantitative CGH analysis reported 6–21% *FGFR1* amplifications in cohorts of 32 and 70 ESCC [24,25]. However, our finding of 8.9% *FGFR1* amplification in ESCC is almost identical to a recent FISH study on Asian ESCC, reporting 8.6% amplification using the same threshold (ratio ≥ 2.0) for amplification [26]. Data from The Cancer Genome Atlas (TCGA https://tcga-data.nci.nih.gov/tcga) on esophageal carcinomas (September 2015) indicate *FGFR1* amplification in 11.1% of 45 squamous cell carcinomas with data on copy number alterations, which is also well in line with 8.9% in our study. That no FGFR1 amplification was reported in 25 adenocarcinomas further supports the concept of marked differences in the FGFR1 amplification frequencies between these two histological subtypes. TCGA data, moreover, suggest that *FGFR1* mutations are rare events (< 2%) in this tumor type. It is, therefore, likely that the putative oncogenic function of amplified *FGFR1* is typically mediated by the wild type gene.

Comparison of FGFR1 mRNA expression levels in a small set of *FGFR1* amplified and non-amplified cancers revealed a wide range of expression levels in both subgroups. An overall high *FGFR1* expression level in amplified cancers was mainly driven by two *FGFR1* amplified cancer with particularly high mRNA expression levels. These findings suggest that gene amplification is one important mechanism for FGFR1 overexpression but also indicates that other mechanisms can lead to a significant up-regulation of FGFR1 expression.

We found a striking predominance of *FGFR1* amplification in squamous cell cancers (8.9%) as compared to adenocarcinomas (1.6%) in our study on 510 esophageal cancers. A higher prevalence of *FGFR1* amplification in ESCC as compared to EADC had also been suggested in a previous study comparing 70 ESCC and 189 EADC [25]. In addition, differences in the *FGFR1* amplification rate between squamous cell carcinoma and adenocarcinomas has also been reported from cancers of the lungs [35,36]. These findings suggest a particular role of *FGFR1* activation for the development of a squamous cell phenotype. It is possible, that this finding is linked to specific mutagenic agents such as cigarette smoke. It is well known that squamous cell cancers of the esophagus and lungs are linked to smoking [37–39]. Differences in the amplification frequency between ESCC and EADC have also been reported from other genes, including SOX2, PIK3CA, MYC, CCND1, which had a higher amplification frequency in ESCC, and GATA4 as well as GATA6, which had a higher amplification frequency in EADC [25]. Of note, many of these genes are transcription factors. It is, thus, tempting to speculate that amplification and overexpression of these genes results in activation of specific genetic programs that favor development of the one ore the other histological subtype of esophageal carcinomas. In fact, amplification of GATA4 and GATA6 is often found in adenocarcinomas from other origins [25,40].

An early role of *FGFR1* activation for squamous cell phenotype development is supported by our analysis of ESCC precursor lesions. It can be expected that molecular events arising before or during malignant transformation should be present in all cancer cells of the resulting tumor bulk. We found *FGFR1* amplification in six of eight samples of squamous cell dysplasia adjacent to *FGFR1* amplified cancers, and 15 of 18 *FGFR1* amplified ESCC showed homogeneous amplification. These findings, despite the low number of cases, might suggest that *FGFR1* amplification is an early event in ESCC. A tumor-initiating role of *FGFR1* is also supported by studies from other cancer types. For example, *FGFR1* amplification was found in in-situ carcinomas and low-grade ER positive breast cancers [41,42] and in early stage lung cancers [35].

Only recently, FGFR1 has gained considerable interest as a target for gene specific therapies. A multitude of selective and non-selective small molecule inhibitors targeting FGFR1 and related tyrosine kinases are currently under investigation in preclinical and clinical trials, including the non-selective inhibitors dovitinib, Ki23057, and ponatinib, and the highly selective inhibitors AZD4547 and BGJ398. Preclinical studies have demonstrated the efficacy of AZD4547 and BGJ398 on *FGFR* gene—amplified cancers both in cell line and mouse models [43–47]. In a phase II clinical trial (NCT01795768), AZD4547 showed therapeutic efficacy as a second-line treatment in patients with *FGFR1*- and *FGFR2*-amplified breast cancer, squamous cell carcinoma of the lung, or gastro-esophageal adenocarcinoma. In addition, one pre-clinical study suggests that such treatment may also hold promise for esophageal cancer [48]. The lack of relevant intratumoral heterogeneity of *FGFR1* amplifications in our study suggests that anti-FGFR1 therapies may be effective in esophageal cancers harboring this alteration, and encourages future clinical trials in *FGFR1* amplified ESCC.

*FGFR1* amplification was unrelated to tumor stage, grade, lymph node metastasis and clinical outcome in the 202 esophagus squamous cell carcinomas analyzed in this study. Additional data on the clinical relevance of *FGFR1* alterations in ESCC are only available from Asian patients. Two studies on 526 Korean and 79 Japanese patients report associations between *FGFR1* amplification [26] and immunohistochemical overexpression [49] and shorted overall survival. It is possible that ethnical differences between Caucasian and Asian patients might account for the discrepant findings. Such ethnical differences have been described for various relevant molecular cancer features, including *HER2* amplification in breast cancer [50], *TMPRSS2-ERG* gene fusion in prostate cancer [51], and *MET* mutation in lung cancer [52].

In this study, a tissue microarray composed from a single 0.6 mm punch per tissue sample was used. We have previously shown that using multiple cores (e.g. 3 cores per tissue spot) does not necessarily increase the ability to identify associations of biomarkers with tumor phenotype and prognosis but has always the disadvantage of additional work and tissue requirements [53]. Using multiple cores can be useful to increase the number of analyzable cancers but can lead to statistical problems if unequal amounts of tissue are analyzed per tumor. In fact, there is a large number of studies using TMAs with one 0.6 mm cores that confirm the known prognostic relevance of virtually all previously established clinically useful biomarkers, for instance, between alterations of HER2 [54] or p53 [55] and survival in breast cancer, between vimentin expression and prognosis in kidney cancer [56], and even between heterogeneous markers such as Ki67 labeling index and prognosis in urinary bladder cancer [57], breast cancer [58] and prostate cancer [53].

Data from The Cancer Genome Atlas (TCGA) on 70 esophageal carcinomas (45 ESCC and 25 EADC) suggest that *FGFR1* mutations are rare events (< 2%) in this tumor type. It is, therefore, likely that the putative oncogenic function of *FGFR1* is typically mediated by the wild type gene.

In summary, the results of our study provide strong evidence that *FGFR1* amplification is an early molecular event linked to the squamous cell subtype of esophageal cancers. The high homogeneity and high level of *FGFR1* amplification argues for FGFR1 representing a promising drug target in ESCC.

## Supporting Information

S1 Table *FGFR1* gene copy number alterations. Legend S1. Polyploidy: ratio >0.8 but <1.2 and more than two *FGFR1* copies, Gain: ratio ≥1.2 but < 2.0.
(TIF)
