# Supplementary figures and images for: FGFR1 Amplification Is Often Homogeneous and Strongly Linked to the Squamous Cell Carcinoma Subtype in Esophageal Carcinoma
Source: PLoS One. 2015 Nov 10;10(11):e0141867. doi: 10.1371/journal.pone.0141867 (PMC4640518; doi:10.1371/journal.pone.0141867)

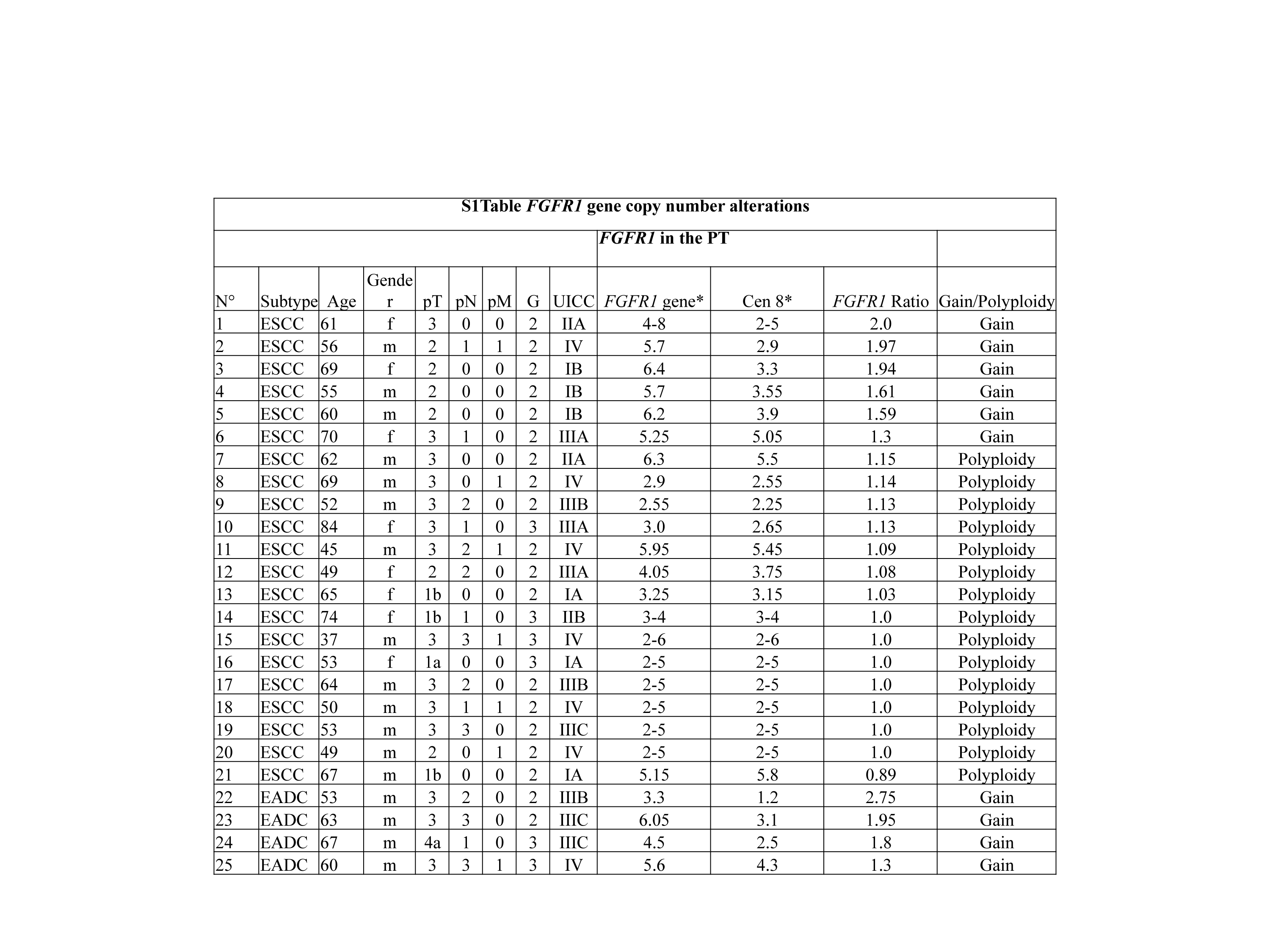

Supplement: S1 Table — Legend S1. Polyploidy: ratio >0.8 but <1.2 and more than two FGFR1 copies, Gain: ratio ≥1.2 but < 2.0. (TIF) [file pone.0141867.s001.tif]
